# Anti‐Cancer Effect of a New 5‐FU Derivative Containing Triazole‐Bearing Mannose (5‐FUD‐MAN) Against Human Breast Cancer Cells Through LC3B‐Mediated Cell Death

**DOI:** 10.1002/jbt.70806

**Published:** 2026-03-29

**Authors:** Ebru Şanci, Azada Aliyeva, Buket Bakan, Mustafa Özkaraca, Erkan Halay, Kadir Ay, Tamer Karayildirim, N. Ulku Karabay Yavasoglu

**Affiliations:** ^1^ Center for Drug Research & Development and Pharmacokinetics Applications Ege University Izmir Turkey; ^2^ Faculty of Science, Department of Molecular Biology and Genetics Atatürk University Erzurum Turkey; ^3^ Faculty of Veterinary, Department of Pathology Cumhuriyet University Sivas Turkey; ^4^ Banaz Vocational School, Chemical Technology and Laboratory Technology Usak University Usak Turkey; ^5^ Faculty of Letter and Science, Department of Chemistry Manisa Celal Bayar University Manisa Turkey; ^6^ Faculty of Science, Department of Chemistry Ege University Izmir Turkey; ^7^ Faculty of Science, Department of Biology Ege University Izmir Turkey

**Keywords:** 1,2,3‐triazole, 5‐fluorouracil, apoptosis, autophagy, breast cancer, mannose

## Abstract

Breast cancer remains one of the most common malignancies affecting women worldwide. Despite the effectiveness of traditional chemotherapeutic agents such as 5‐fluorouracil (5‐FU), their lack of selectivity often results in damage to healthy tissues, leading to undesirable adverse effects. The aim of this study was to modify 5‐FU with mannose containing 1,2,3‐triazole compound to reduce its toxic effect, and to investigate the anticancer properties of the resulting 5‐FU derivative (5‐FUD‐Man) in ER‐positive MCF‐7 breast cancer cells. Our results demonstrated that while 5‐FU caused significant cytotoxicity in both cancerous and healthy cells, 5‐FUD‐Man showed selective cytotoxicity, with minimal effects on MCF‐10A cells. Furthermore, immunofluorescence staining results indicated that 5‐FUD‐Man was more strongly activated apoptosis (Caspase‐3, AIF), autophagy‐mediated (LC3B), and stress‐associated signaling pathways (ERK1/2) in MCF‐7 cells compared to 5‐FU. These findings suggest that the combined use of carbohydrate‐based targeting via mannose and a bioactive triazole compound may enhance the selectivity and therapeutic efficacy of 5‐FU‐based treatments in breast cancer. Overall, 5‐FUD‐Man appears to be a promising candidate for further development as a more targeted and potentially safer therapeutic strategy.

## Graphical abstract

The anticancer activity of 5‐FU modified with mannose containing 1,2,3‐triazole compound in MCF‐7 breast cancer cells were revealed in this study. While 5‐FU caused cytotoxicity in both cancerous and healthy cells, 5‐FUD‐Man showed selective cytotoxicity. Immunofluorescence staining results indicated that 5‐FUD‐Man was more strongly activated LC3B dependent autophagic cell death in MCF‐7 cells compared to 5‐FU.

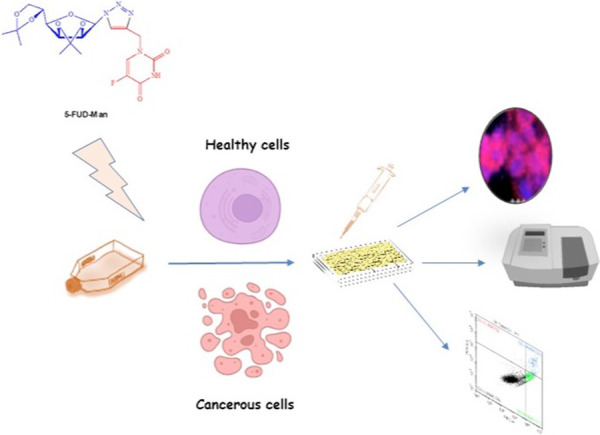

## 1 Introduction

Breast cancer is the most prevalent cancer among women and one of the most aggressive malignancies worldwide. Its initiation and progression are influenced by both genetic and environmental factors [1, 2]. Elevated circulating estrogen levels and dysregulation of estrogen signaling pathways play a central role in breast cancer development [3]. Consequently, many therapeutic strategies target the estrogen receptor (ER) signaling pathway. In addition to hormone therapies, various chemotherapeutic agents including doxorubicin, paclitaxel, carboplatin, and 5‐fluorouracil (5‐FU) are widely used in clinical management [4]. Among them, 5‐FU is a first‐line cytotoxic drug used in the systemic treatment of multiple solid tumors [5]. However, its nonspecific toxicity remains a major limitation, as it damages both cancerous and healthy cells. Understanding the mechanism of action of 5‐FU has driven efforts to enhance its anticancer efficacy and reduce its side effects. In recent years, significant progress has been made in the design of 5‐FU derivatives with improved selectivity and reduced toxicity [6, 7, 8]. In particular, sugar‐conjugated 5‐FU derivatives have emerged as promising candidates because of their potential to enhance tumor targeting, decrease systemic toxicity, and improve accumulation in cancerous tissues.

Carbohydrate‐based drug delivery systems offer several advantages such as protection against enzymatic degradation, controlled drug release, and reduced off‐target effects [9, 10, 11]. These systems represent a promising strategy for improving the bioavailability and safety profile of 5‐FU [12]. In addition, the incorporation of a 1,2,3‐triazole ring, an aromatic five‐membered heterocyclic structure containing three nitrogen atoms, into drug design has gained considerable attention because of its remarkable chemical stability and diverse biological activity [13, 14]. Triazoles possess both hydrogen bond donor and acceptor capabilities, enabling strong interactions with biological targets. Their versatility allows the development of compounds with a wide range of pharmacological properties, including anticancer, antibacterial, antiviral, antifungal, anti‐tubercular, and analgesic activities [15, 16]. Furthermore, triazoles are frequently used in polymeric carriers and functionalized nanoparticles due to their aqueous solubility and chemical compatibility [17, 18].

This study aimed to investigate the selective effects of 5‐FU and a novel 5‐FU derivative modified with mannose (5‐FUD‐Man) and 1,2,3‐triazole on both healthy and cancerous breast cells. Specifically, the cytotoxicity mechanisms and oxidative potentials of these compounds were evaluated in MCF‐7 cells, an ER‐positive, progesterone receptor (PR)‐positive, and human epidermal growth factor receptor 2 (HER2)‐negative breast cancer cell line, and MCF‐10A, a non‐tumorigenic breast epithelial cell line. The study focused on elucidating how these compounds modulate cell death pathways at the mechanistic level.

## 2 Material and Methods

### 2.1 Materials

**FIGURE 1:**
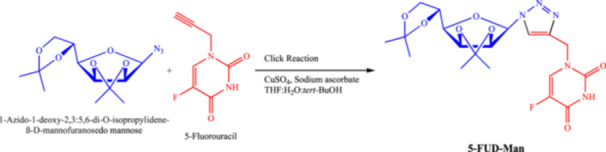
Synthesis routes and IUPAC names of synthesized compound.

**FIGURE 2:**
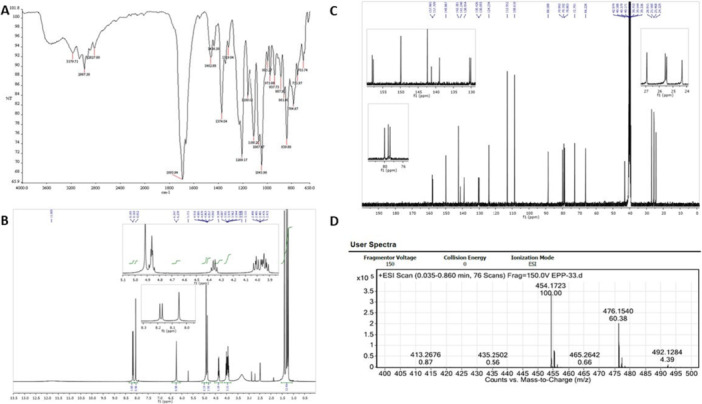
(A) FT‐IR Spectrum (B) ^1^H NMR Spectrum in DMSO‐*d*_*6*_, (C) ^13^C NMR Spectrum in DMSO‐*d*_*6*_, and (D) HRMS Spectrum.

1‐[{1′‐(2″,3″:5″,6″‐Di‐O‐isopropylidene‐β‐d‐mannofuranosyl)−1′H‐1′,2′,3′‐triazol‐4′‐yl}methyl]‐5‐fluorouracil contains triazole‐bearing mannose as carbohydrate and 5‐fluorouracil as nucleobase. It was synthesized according to the method described by Halay et al. (2018) [8] and is referred to as 5‐FUD‐Man in the rest of the manuscript for ease of reading. The chemical structures and synthesis routes of 5‐FUD‐Man are presented in Figure 1. The FT‐IR, ^1^H NMR, ^13^C NMR and MS Spectra of the compound are shown in Figure 2.

### 2.2 Cell Culture and Cytotoxicity Assay

MCF‐10A (Human breast epithelial cell line, ATCC‐CRL‐10317) and MCF‐7 (Human breast epithelial cancer cell line, ATCC‐HTB‐22) cells were cultured in Dulbecco′s modified eagle medium–high glucose (DMEM) supplemented with 10% fetal bovine serum (FBS), 100 U/ml penicillin/streptomycin at 37°C in 5% CO_2_ incubation. The culture medium was changed every 2 days. The cytotoxicity test was performed with WST‐1 assay which was related to the metabolic activation of cells [19]. Cells at a concentration of 2 × 10^4^ cells/mL were seeded with 100 μL each in 96‐well plates and incubated for 24 h at 37°C, under 5% CO_2_. The cultured cells were treated with different concentrations (6.25–500 μg/mL) of 5‐FUD‐Man for 24 h exposure time at 37°C. 5‐fluorouracil (5‐FU) was used as positive control. Morphological changes of the cells were observed with inverted light microscope and optical density measured in a multiwell plate reader (THERMO microplate reader) at 450 nm absorbance. All compounds were tested in triplicate. The relative viability was calculated against untreated cells as control and presented as percentage. The cell viability (%) was calculated as the formula below;

$$\mathrm{Viable}\;\mathrm{cells}\;\left(\%\right)=\frac{\left(\mathrm{absorbance}\;\mathrm{of}\;\mathrm{the}\;\mathrm{treated}\;\mathrm{cells}\right)-(\mathrm{absorbance}\;\mathrm{of}\;\mathrm{the}\;\mathrm{blank})}{\left(\mathrm{absorbance}\;\mathrm{of}\;\mathrm{the}\;\mathrm{control}\right)-(\mathrm{absorbance}\;\mathrm{of}\;\mathrm{the}\;\mathrm{blank})}\;\times 100$$

### 2.3 Flow Cytometry Analysis

Cells were seeded in 96‐well plates at a density of 1 × 10^4^ cells per well and allowed to adhere and proliferate for 24 h prior to treatment. Subsequently, cells were exposed to various concentrations of 5‐FUD‐Man (125–500 µg/mL), as determined from the cytotoxicity assay, and incubated for an additional 24 h. Following treatment, the culture supernatant from each well, containing nonadherent cells, was collected into pre‐labeled centrifuge tubes. Adherent cells from the same wells were then trypsinized and combined with their corresponding supernatant to ensure inclusion of all cell populations. The collected cell suspensions were centrifuged, and the supernatant was discarded. The resulting pellets were resuspended in Annexin V binding buffer containing Annexin V‐FITC and propidium iodide (PI) dyes and incubated for 30 min at room temperature in the dark. Flow cytometric analysis was performed using a flow cytometer (Beckman Coulter CytoFLEX) to determine the percentages of viable, apoptotic (programmed cell death), and necrotic (cell death due to external stimuli) cells within the population.

### 2.4 Determination of Oxidative Stress Formation

The DCFH‐DA and DHE assays are fluorescence‐based methods used to evaluate oxidative stress through the detection of reactive oxygen species (ROS). These assays rely on the oxidation of the fluorescent molecular probes 2′,7′‐dichlorodihydrofluorescein diacetate (DCFH‐DA) and dihydroethidium (DHE) by different ROS species [20]. DCFH‐DA freely permeates cell membranes and is hydrolyzed by intracellular esterases to form non‐fluorescent DCFH, which is subsequently oxidized to fluorescent 2′,7′‐dichlorofluorescein (DCF) in the presence of hydroxyl radicals. DHE, on the other hand, is oxidized to fluorescent 2‐hydroxyethidium (2‐OH‐E⁺) in the presence of superoxide radicals, allowing accurate measurement of DHE fluorescence with minimal interconversion variability. In this study, concentration‐dependent measurements of ROS formation were performed in MCF‐10A and MCF‐7 cells treated with various concentrations of 5‐FUD‐Man (6.25–500 µg/mL) and incubated at 37°C for 4 h. Hydrogen peroxide‐treated cells (100 µM H_2_O_2_) were used as positive controls, while untreated cells served as negative controls. After exposure, cells were washed three times with PBS to minimize background interference and subsequently stained with 20 µM DCFH‐DA and 20 µM DHE for 30 min at 37°C. Following staining, cells were washed twice with PBS, and fluorescence intensity was measured using a microplate reader at an excitation wavelength of 485 nm and an emission wavelength of 535 nm. Data were expressed as the percentage of fluorescence relative to the corresponding negative control.

### 2.5 Double‐Label Immunofluorescence Investigation

After 24 h of exposure to 5‐FU and 5‐FUD‐Man at various concentrations (125, 250, and 500 μg/mL) determined from cytotoxicity data, MCF‐10A and MCF‐7 cells were fixed with methanol for 5 min at −20°C and subsequently washed with PBS (1×). The fixed cells were then permeabilized with PBS containing 0.1% Triton X‐100 (Merck Millipore, Catalog No. 1.08603.1000) for 15 min at room temperature. Following permeabilization, the cells were washed and incubated with PBS containing 2% BSA for 60 min at room temperature. After blocking, the cells were incubated overnight at 4°C with primary antibodies against cleaved Caspase‐3 (Elabscience, Catalog No. E‐AB‐30004), LC3B (Santa Cruz Biotechnology, Catalog No. sc‐271625), AIF (Santa Cruz Biotechnology, Catalog No. Bs‐0037R), and ERK1/2 (Affinity Biosciences, Catalog No. AF1015), each diluted 1:200 in PBS containing 1% BSA. The following day, cells were washed with PBS and incubated with secondary antibodies, goat anti‐rabbit FITC (Jackson ImmunoResearch, Catalog No. 111‐095‐003) and goat anti‐mouse IgG Texas Red (Novus Biologicals, Catalog No. NBP1‐73623), both diluted 1:50 in PBS. Cells were then incubated for 1 h at room temperature in the dark.

Finally, DAPI (Abcam, Catalog no. ab228549) was applied to the washed cells and examination was performed by using fluorescence microscopy (Zeiss Axio). Fluorescence intensity was quantified using the Fiji ImageJ software (NIH, USA).

### 2.6 Statistical Analysis

Statistical analyses of cytotoxicity and ROS formation data were performed using GraphPad Prism 9. One‐way analysis of variance (ANOVA) followed by Tukey test was used for comparison between the groups. Data were expressed as the mean ± standard deviation (SD). Statistical analyses of immunofluorescence data were performed using the SPSS software version 25.0 (IBM Corp., Armonk, New York, USA). One‐way ANOVA followed by Duncan's multiple comparison test was used for comparison between the groups. The experiments were conducted in triplicate, and the results were expressed as mean ± SD, and Values of *p* ≤ 0.05 were regarded as statistically significant.

## 3 Results

### 3.1 Cytotoxicity Test Results

**FIGURE 3:**
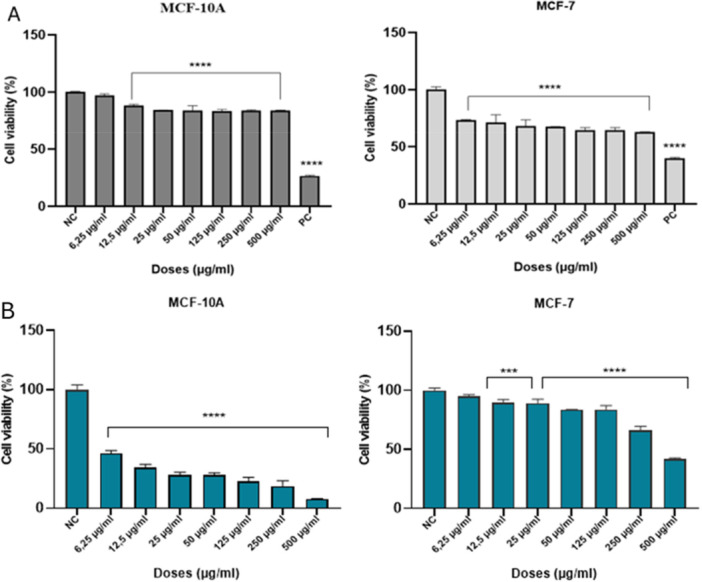
Cytotoxicity test results of 5‐FUD‐Man (A) and 5‐FU (B) at different concentrations in MCF‐10A and MCF‐7 cells after 24‐h exposure by WST‐1 assay. The experiments were conducted in triplicate. One‐way ANOVA followed by Tukey test was used for comparison between the groups. Data are presented as mean ± SD for three independent experiments. (PC: 5‐FU). (*indicates the difference compared to the negative control group). ***p < 0.001; ****p < 0.0001.

The cytotoxic effects of 5‐FUD‐Man and 5‐FU were evaluated in both healthy (MCF‐10A) and cancerous (MCF‐7) cells across a range of concentrations following 24 h of exposure (Figure 3). The results demonstrated that 5‐FUD‐Man induced a concentration‐dependent decrease in cell viability. Notably, 5‐FUD‐Man exhibited minimal cytotoxicity toward MCF‐10A cells after 24 h, with cell viability remaining comparable to that of the negative control group. In contrast, 5‐FUD‐Man significantly reduced the viability of MCF‐7 cells, with higher concentrations inducing cell death approaching those observed in the positive control. Conversely, while 5‐FUD‐Man had little effect on healthy MCF‐10A cells (Figures 3A), 5‐FU exhibited marked cytotoxicity in this cell line, resulting in a pronounced decrease in cell viability (Figure 3B). In the MCF‐7 cancer cells, 5‐FU also decreased cell viability in a concentration‐dependent manner; however, its anticancer effect was less pronounced compared to that of 5‐FUD‐Man.

### 3.2 Flow Cytometry Analyses Results

**FIGURE 4:**
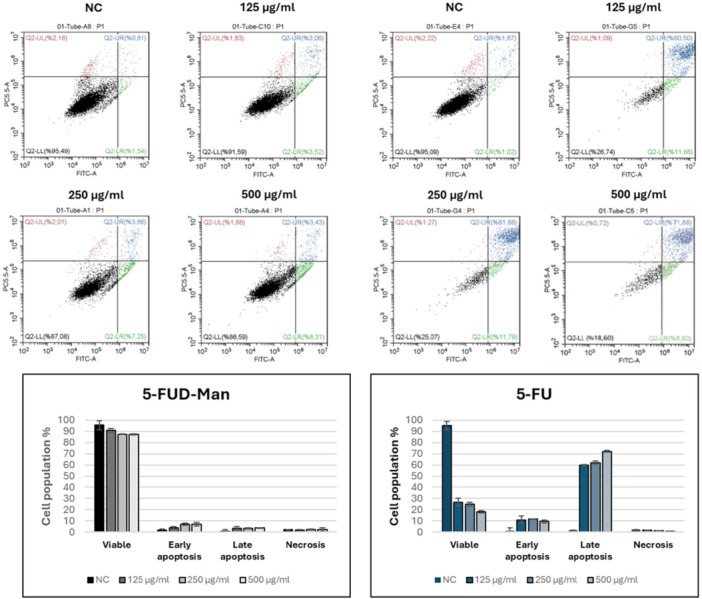
Apoptotic activities of 5‐FU and 5‐FUD‐Man on MCF‐10A cell line. Flow cytometry imaging of compounds (above), and the percent cell population as graphs (below). The experiments were conducted in triplicate.

**FIGURE 5:**
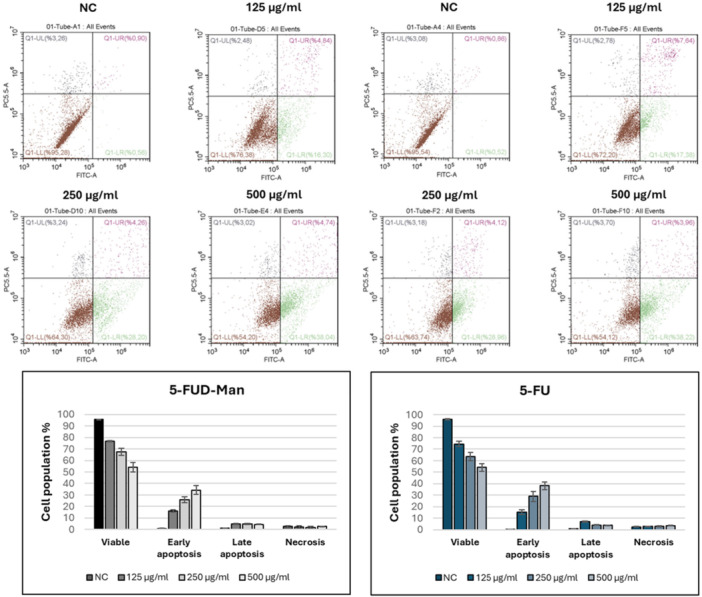
Apoptotic activities of 5‐FU and 5‐FUD‐Man on MCF‐7 cell line. Flow cytometry imaging of compounds (above), and the percent cell population as graphs (below). The experiments were conducted in triplicate.

Flow cytometry analysis results of 5‐FU and 5‐FUD‐Man on MCF‐10A and MCF‐7 cell lines are presented in Figures 4 and 5, respectively. For both compounds, cell viability decreased with increasing concentrations, accompanied by a corresponding increase in apoptotic cell populations. Notably, 5‐FUD‐Man did not induce apoptosis in healthy MCF‐10A cells.

### 3.3 ROS Formation

**FIGURE 6:**
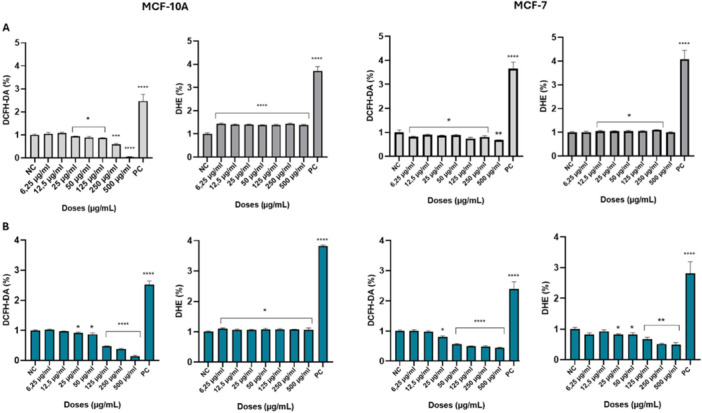
Effects of 5‐FUD‐Man (A) and 5‐FU (B) on ROS level in MCF‐10A and MCF‐7 cell lines after 4 h incubation. Cells were stained with 20 µM DCFH‐DA and 20 µM DHE dye. NC, negative control (not treated with H_2_O_2_); PC, positive control (treated with H_2_O_2_). Data represents the mean fluorescence signal with ±SD with calculation against the negative control and at least 10,000 events were collected per sample. The experiments were conducted in triplicate. One‐way ANOVA followed by Tukey test was used for comparison between the groups. (* indicates the difference compared to the negative control group) * *p* < 0.05; **p < 0.01; ***p < 0.001; ****p < 0.0001.

Treatment of MCF‐10A cells with 5‐FUD‐Man and 5‐FU, particularly at higher concentrations, resulted in a statistically significant decrease in the DCFH‐DA fluorescence signal compared to the negative control group, indicating reduced general reactive oxygen species (ROS) levels. In contrast, the DHE fluorescence signal was significantly increased for both compounds (Figure 6, left side). Similarly, treatment of MCF‐7 cells with different concentrations of 5‐FUD‐Man and 5‐FU produced results comparable to those observed in MCF‐10A cells for the DCFH‐DA fluorescence signal. However, in MCF‐7 cells, 5‐FUD‐Man treatment induced an increase in the DHE signal relative to the negative control, whereas 5‐FU treatment caused a decrease (Figure 6, right side).

### 3.4 Double‐Label Immunofluorescence Investigation

**FIGURE 7:**
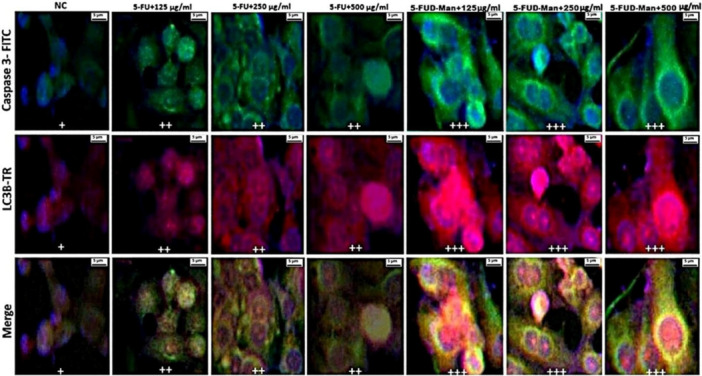
In the MCF‐7 cell line, Caspase 3 and LC3B positivity was observed as follows: mild (+) in the NC group, moderate (++) the 125, 250, and 500 µg/mL of 5‐FU treatment groups, and severe (+++) in the 125, 250, and 500 µg/mL of 5‐FUD‐Man treatment groups. Bar: 5 µm; FITC, fluorescein isothiocyanate; TR, Texas Red.

**FIGURE 8:**
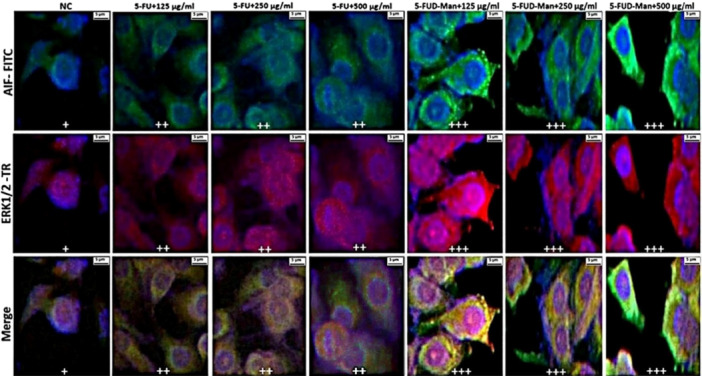
In the MCF‐7 cell line, AIF and ERK 1/2 positivity was observed as follows: mild (+) in the NC group, moderate (++) in the 125, 250, and 500 µg/mL of 5‐FU treatment groups, and severe (+++) in the 125, 250, and 500 µg/mL of 5‐FUD‐Man treatment groups. Bar: 5 µm; FITC, fluorescein isothiocyanate; TR, Texas Red.

**FIGURE 9:**
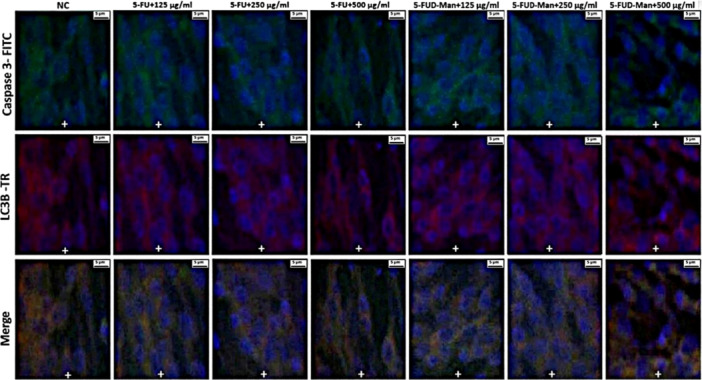
In the MCF‐10A cell line, Caspase 3 and LC3B positivity were observed as mild (+) in all groups. Bar: 5 µm; FITC, fluorescein isothiocyanate; TR, Texas Red.

**FIGURE 10:**
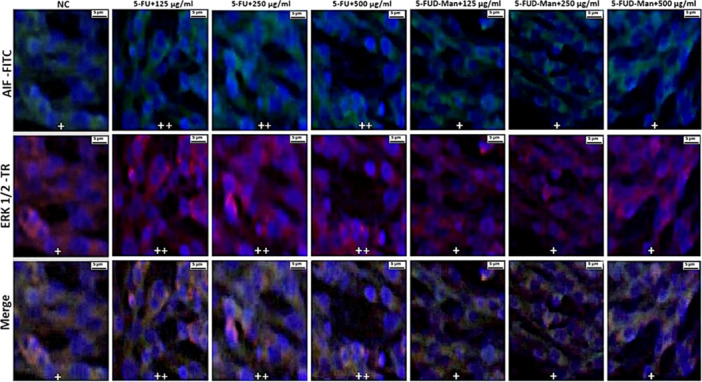
In the MCF‐10A cell line, AIF and ERK 1/2 positivity was observed as follows: mild (+) in the NC, 125, 250, and 500 µg/mL of 5‐FUD‐Man treatment groups, moderate (++) in the 125, 250, and 500 µg/mL of 5‐FU treatment groups. Bar: 5 µm; FITC, fluorescein isothiocyanate; TR, Texas Red.

Statistically significant differences in double immunofluorescence positivity for Caspase‐3, LC3B, AIF, and ERK1/2 were observed among the treatment groups in MCF‐7 cells (Table 1). In contrast, no significant differences were detected among the groups in MCF‐10A cells (Table 2). In the MCF‐7 cell line, immunofluorescence images revealed that the expression levels of Caspase‐3, LC3B, AIF, and ERK1/2 were mild level in the negative control (NC) group, moderate level in the 125, 250, and 500 µg/mL 5‐FU treatment groups, and strong in the 125, 250, and 500 µg/mL 5‐FUD‐Man treatment groups (Figures 7 and 8). Conversely, in MCF‐10A cells, Caspase‐3 and LC3B staining showed no significant positivity. AIF and ERK1/2 exhibited mild expression in the NC and all 5‐FUD‐Man treatment groups, whereas moderate expression was observed in all 5‐FU treatment groups, with statistically significant differences compared to the NC group (Figures 9 and 10).

**TABLE 1:** The intensity of fluorescence positivity on MCF‐7 cell line.

	Intensity of fluorescence positivity			
Groups	Caspase 3	LC3B	AIF	ERK 1/2
NC	21.14 ± 3.28^a^	24.13 ± 5.02^a^	19.24 ± 2.57^a^	22.94 ± 3.87^a^
5‐FU				
125 µg/mL	32.83 ± 6.01^b^	35.83 ± 6.71^b^	31.39 ± 5.13^b^	36.61 ± 4.91^b^
250 µg/mL	33.47 ± 5.04^b^	34.16 ± 3.91^b^	29.36 ± 4.55^b^	37.06 ± 7.25^b^
500 µg/mL	34.20 ± 5.46^b^	36.41 ± 4.49^b^	32.71 ± 3.86^b^	35.69 ± 6.48^b^
5‐ FUD‐Man				
125 µg/mL	96.24 ± 9.17^c^	100.15 ± 6.20^c^	96.25 ± 8.81^c^	94.17 ± 7.35
250 µg/mL	99.02 ± 6.31^c^	103.21 ± 15.48^c^	95.24 ± 9.03^c^	98.04 ± 6.13
500 µg/mL	98.17 ± 8.54^c^	99.08 ± 9.61^c^	98. 02 ± 8.34^c^	97.22 ± 9.19

*Note:* One‐way ANOVA followed by Duncan's multiple comparison test was used for comparison between the groups. The experiments were conducted in triplicate. Different letters (a, b, c) in the same column indicate statistical differences between the groups (*p* < 0.001).

**TABLE 2:** The intensity of fluorescence positivity on MCF‐10A cell line.

Groups	Intensity of fluorescence positivity			
	Caspase 3	LC3B	AIF	ERK 1/2
NC	18.31 ± 3.24^a^	16.29 ± 2.14^a^	19.46 ± 3.31^a^	24.13 ± 5.16^a^
5‐FU				
125 µg/mL	21.34 ± 2.42^a^	18.04 ± 3.06^a^	33.54 ± 3.58^b^	30.12 ± 2.28^b^
250 µg/mL	17.34 ± 4.26^a^	19.21 ± 5.61^a^	32.16 ± 2.05^b^	29.90 ± 3.71^b^
500 µg/mL	20.56 ± 3.36^a^	17.45 ± 7.18^a^	31.70 ± 4.09^b^	28.01 ± 2.46^b^
5‐FUD‐Man				
125 µg/mL	21.09 ± 4.47^a^	20.89 ± 5.01^a^	18.46 ± 3.50^a^	21.21 ± 5.01^a^
250 µg/mL	19.82 ± 3.01^a^	22.16 ± 2.49^a^	19.05 ± 3.24^a^	22.65 ± 4.66^a^
500 µg/mL	19.30 ± 4.14^a^	23.01 ± 3.26^a^	22.94 ± 2.11^a^	18.91 ± 2.07^a^

*Note:* One‐way ANOVA followed by Duncan's multiple comparison test was used for comparison between the groups. The experiments were conducted in triplicate. Different letters (a, b, c) in the same column indicate statistical differences between the groups (*p* < 0.001).

## 4 Discussion

The International Agency for Research on Cancer (IARC) and its collaborators recently assessed the current and projected global burden of female breast cancer through an analysis of data from nearly 50 countries. Their findings indicate that, on average, 1 in 20 women worldwide will be diagnosed with breast cancer during their lifetime. If current trends continue, it is estimated that by 2050 there will be approximately 3.2 million new breast cancer cases and 1.1 million breast cancer‐related deaths annually [21]. Consequently, early detection and effective treatment are critical for improving survival rates and quality of life for patients. Advances in medical research have led to the development of targeted therapies, including hormone therapy and immunotherapy, which aim to inhibit cancer cell growth while minimizing damage to surrounding healthy tissues. Although these treatments have significantly improved clinical outcomes, they are associated with several limitations and potential side effects that must be carefully considered for each patient. For instance, hormone therapy is not effective for all breast cancer subtypes and may cause adverse effects with long‐term use. Similarly, targeted therapies can be highly effective against specific cancer subtypes but are not universally applicable and may be associated with high treatment costs. Therefore, continued research is essential to develop more effective, selective, and less invasive therapeutic strategies for breast cancer patients [22].

Triazole‐based compounds have demonstrated diverse anticancer mechanisms, including the inhibition of tyrosine kinases, BET proteins, microtubules, aromatase, and poly(ADP‐ribose) polymerase (PARP). In recent years, triazole‐containing hybrid molecules have attracted considerable attention because of their potent inhibitory effects against various cancer cell lines [15]. The strategy of combining the triazole scaffold with other anticancer pharmacophores has emerged as an innovative approach for developing novel therapeutic agents with improved efficacy and reduced toxicity [23]. Although the anticancer activity of triazole derivatives has been widely investigated over the past decade [24, 25, 26, 27], studies specifically focusing on 1,2,3‐triazole related to 5‐FU remain limited [28, 29, 30]. 5‐FU enters cells through the uracil transport system and is metabolized into active compounds, including fluorodeoxyuridine monophosphate (FdUMP), fluorodeoxyuridine triphosphate (FdUTP), and fluorouridine triphosphate (FUTP). Its anticancer activity primarily results from inhibition of thymidylate synthase, disruption of RNA synthesis, and induction of DNA damage during the S phase of the cell cycle [31]. Although the principal mechanism of 5‐FU involves interference with DNA synthesis and mRNA translation, its cytotoxic effects are not restricted to tumor cells and may also affect healthy tissues [32, 33]. In our study, 5‐FU exhibited strong cytotoxicity in MCF‐10A cells, which represent healthy mammary epithelial cells, whereas 5‐FUD‐Man showed no significant cytotoxic effects on these cells. Both compounds, however, induced concentration‐dependent cytotoxicity in MCF‐7 cancer cells. Consistent with our findings, Cai et al. (2014) reported that 5‐FU inhibits the growth of MCF‐7 cells in a concentration‐dependent manner [34], and Ge et al. (2014) demonstrated its antitumor activity and inhibitory effects on cell proliferation in this cell line [35]. These results suggest that modification of 5‐FU with carbohydrates and 1,2,3‐triazoles not only enhances cytotoxicity against cancer cells but also reduces unwanted effects on healthy cells. In particular, mannose modification appears to increase selective cytotoxicity toward cancer cells. Since cancer cells exhibit higher glucose uptake, they can preferentially internalize carbohydrate‐modified 5‐FU compared to healthy cells [36]. Additionally, the intrinsic anticancer properties of 1,2,3‐triazoles are likely to contribute to the enhanced cytotoxic activity of 5‐FUD‐Man against cancer cells [17].

Reactive oxygen species (ROS) play a critical role in regulating cellular metabolism under both physiological and pathological conditions. Physiological ROS production is essential for the spatial and temporal modulation of key cellular processes, including proliferation, signaling, apoptosis, and senescence. Consequently, accurate and reproducible quantification of ROS is vital for understanding cellular homeostasis. Fluorescence‐based assays are commonly employed to characterize intracellular ROS, with DCFH‐DA being among the most widely used probes. DCFH‐DA staining is a simple and cost‐effective approach for detecting ROS in cells. It allows measurement of ROS generation following chemical treatments or genetic modifications, providing valuable insights into oxidative stress and underlying mechanisms [37]. However, this probe has notable limitations. DCFH‐DA, the diacetate derivative of DCFH, readily penetrates cell membranes and is hydrolyzed by intracellular esterases into the non‐fluorescent compound DCFH. This compound is oxidized to the fluorescent product 2′,7′‐dichlorofluorescein (DCF) by various ROS, making it non‐specific for any ROS species. Importantly, DCFH is not directly oxidized by H₂O₂; oxidation occurs only after H₂O₂ is converted into more reactive species via redox‐active metals or heme proteins such as cytochrome c or peroxidases [38]. Therefore, DCFH‐DA is not suitable for accurate evaluation of intracellular H₂O₂ levels. DCFH‐DA can also generate intermediate products (DCFH⁻/DCF) that, in the presence of oxygen, produce additional superoxide radicals. The subsequent dismutation of O₂⁻ generates further H₂O₂, potentially leading to artifactual increases in fluorescence intensity and overestimation of ROS levels. In contrast, DHE reacts specifically with the superoxide radical to form the stable fluorescent product 2‐hydroxyethidium (2‐OH E⁺), making it a more reliable probe for quantifying intracellular superoxide, particularly in high‐throughput assays [39]. In the present study, both DCFH‐DA and DHE probes were employed to evaluate ROS levels. Due to the differences in probe specificity and reactivity, distinct ROS profiles were observed with each method. Nevertheless, DHE fluorescence signal was significantly increased for both compounds in MCF10A cells. In MCF‐7 cells, while 5‐FUD‐Man treatment led to an increase in the DHE signal relative to the negative control, whereas 5‐FU treatment resulted in a decrease. It is known that ROS production and antioxidant capacity differ between normal and cancerous cells. Normal cells typically maintain low ROS levels and effective antioxidant defenses, whereas cancer cells often exhibit elevated ROS production, which can contribute to poor prognosis. Modulation of ROS has been proposed as a potential strategy to overcome 5‐FU resistance in cancer therapy [40]. In many cancer cells, increased ROS levels are closely associated with the induction of apoptosis, suggesting that ROS modulation may play a key role in mediating the cytotoxic effects of 5‐FU.

The immunopositivity levels of key proteins such as Caspase‐3, LC3B, AIF, and ERK1/2, involved in distinct cell death mechanisms, were found to be lower in MCF‐10A cells compared to MCF‐7 cells. This indicates that healthy cells exhibit greater resistance to treatment and possess stronger survival mechanisms. In contrast, MCF‐7 cancer cells, characterized by higher proliferation rates and genetic heterogeneity, displayed a more pronounced response to both 5‐FU and 5‐FUD‐Man treatments. This effect was particularly evident in the 5‐FUD‐Man‐treated groups, where apoptosis and autophagy pathways were more effectively activated. Consistent with the cell viability results, the significant increase in the immunopositivity levels of these proteins in MCF‐7 cells suggests a robust activation of cell death mechanisms. The strong therapeutic response of 5‐FUD‐Man against MCF‐7 cells indicates its potential to suppress cancer cell survival signals and accelerate cell death. At higher concentrations, 5‐FUD‐Man induced greater cellular stress and damage compared to 5‐FU, resulting in a more comprehensive activation of apoptotic and autophagic processes in MCF‐7 cells. This enhanced effect was more selective toward cancer cells, with minimal impact on healthy MCF‐10A cells, highlighting 5‐FUD‐Man's potential as a more targeted and potent therapeutic agent against breast cancer. The differential responses observed between healthy and cancerous cells are likely associated with the distinct regulatory mechanisms governing key molecular markers involved in cell death and survival pathways.

Autophagy and apoptosis are fundamental cellular processes that regulate cell death, and there are evidence suggesting they interact with other [41, 42, 43]. Both mechanisms play important roles in tumor suppression: autophagy contributes to the elimination of damaged or malignant cells, while apoptosis prevents the survival of abnormal cells [44, 45]. The membrane‐bound microtubule‐associated protein light chain 3 (LC3) serves as a key indicator of autophagic activity. The conversion of LC3‐I to LC3‐II, regulated by autophagy‐related genes (ATG3, ATG7, and ATG8), is essential for autophagosome formation. Therefore, the lysosomal turnover of the autophagosomal marker LC3‐II reflects autophagic activity, and detection of LC3 through immunoblotting or immunofluorescence has become a reliable approach for monitoring autophagy and related processes, including autophagic cell death [46, 47]. Among the LC3 isoforms (LC3A, LC3B, and LC3C), LC3B is the most widely expressed and most frequently used marker in autophagy studies [48]. LC3B plays a complex and critical role in tumorigenesis, therapeutic resistance, and the regulation of both cell death and survival mechanisms. Autophagic cell death can be categorized into two major forms. The first, autophagy‐dependent cell death (ADCD), includes processes such as excessive mitophagy, autosis, and endoplasmic reticulum (ER)‐phagy, which depend directly on autophagy‐related molecules. The second, autophagy‐mediated cell death (AMCD), involves crosstalk between autophagic machinery and other cell death pathways, whereby autophagy can activate apoptosis, ferroptosis, or necroptosis to facilitate cell demise [49]. The relationship between autophagy and apoptosis is context‐dependent, and they sometimes collaborate and sometimes oppose each other depending on the influencing factors and surrounding conditions. In some times, autophagy combines with apoptosis to promote cell death, such as in chemotherapy‐treated cells. On a molecular level, ATGs, which play a role in autophagosome formation, have been found to be involved in apoptosis. Several ATGs are recognized and cleaved by caspases [42]. Beclin‐1, a key regulator of autophagy, and it is considered a substrate for multiple caspases, including caspase‐3, ‐6, ‐7, ‐8, and ‐9 [49, 50, 51]. Autophagy can either enhance or suppress apoptosis, and conversely, apoptosis can modulate autophagic activity [52, 53]. In addition to this, LC3B has been shown to activate the extrinsic apoptotic pathway through interactions with Fas and Fas ligand (FasL), thereby promoting apoptosis [54]. Moreover, ROS‐induced LC3B expression has been associated with enhanced apoptotic activity, and ROS‐dependent upregulation of LC3B has been shown to suppress tumor growth in vitro [51]. In our study, the increase in LC3B and Caspase 3 expression, particularly on MCF‐7 cancer cells, following 5‐FUD‐Man treatment was interpreted as indicating that ROS‐dependent induced LC3B effectively stimulated apoptotic processes. Additionally, apoptosis‐inducing factor (AIF) plays a central role in caspase‐independent programmed cell death by triggering nuclear apoptosis [55]. Under conditions of cellular stress, AIF translocates from the mitochondrial intermembrane space to the nucleus, where it binds to DNA and promotes fragmentation by endonucleases [56]. In our study, the increased AIF levels observed in MCF‐7 cells following 5‐FUD‐Man exposure further support the notion that this compound promotes cell death through both caspase‐dependent and caspase‐independent pathways.

ERK1/2 activation regulates a broad range of cellular substrates, including transcription factors, kinases, phosphatases, cytoskeletal proteins, apoptosis regulators, and numerous other signaling‐associated molecules that collectively influence cell proliferation and cell death [57]. Although ERK1/2 signaling is primarily associated with promoting cell growth and survival, sustained or excessive activation can also induce cell cycle arrest and apoptosis under certain conditions. In response to oncogenic stimuli, such as receptor tyrosine kinases (RTKs), Ras, or Raf mutations, ERK1/2 activation can trigger growth inhibition in both healthy and tumor cells. This indicates that cells must maintain ERK1/2 activity within a specific signal intensity range to regulate proliferation and survival effectively. Both insufficient and excessive ERK1/2 signaling can activate antiproliferative responses and cell death pathways [58]. Furthermore, ERK1/2 activity has been closely linked to autophagy and autophagy‐related cell death, particularly in non‐neuronal cell models exposed to various stressors, including TNF‐α treatment in MCF‐7 cells. ERK‐dependent autophagic activity is often accompanied by the induction of classical autophagy markers, such as LC3 expressions. Notably, direct activation of ERK through overexpression of constitutively active MEK has been shown to promote autophagy even in the absence of additional stimuli [59, 60, 61]. In the present study, the observed increase in ERK1/2 immunopositivity in MCF‐7 cells following 5‐FU and 5‐FUD‐Man treatments suggests that these compounds induce ERK1/2‐mediated growth inhibition, thereby contributing to the suppression of tumor cell proliferation. Additionally, 5‐FU exhibited strong cytotoxic effects on healthy MCF‐10A cells, as indicated by elevated immunopositivity for AIF and ERK1/2 comparable to that observed in MCF‐7 cells. In contrast, 5‐FUD‐Man treatment did not increase the expression of these proteins in MCF‐10A cells, suggesting a protective effect against undesired cytotoxicity in healthy tissue. Collectively, these findings indicate that 5‐FUD‐Man exerts selective cytotoxic effects on cancer cells while preserving healthy cells, underscoring its potential as a promising candidate for targeted breast cancer therapy.

## 5 Conclusion

This study showed that 5‐FU had strong cytotoxic effect on MCF‐10A cells, while 5‐FUD‐Man had no cytotoxicity on the healthy cells. Both compounds exerted distinct concentration‐dependent cytotoxic effects on cancerous MCF‐7 cells. These findings revealed that using carbohydrates and 1,2,3‐triazoles provided not only better cytotoxic effect of 5‐FU derivative on cancer cells but also protected healthy cells to unwanted cytotoxic side effect. 5‐FUD‐Man induced greater cellular damage and stress than 5‐FU, leading to a more comprehensive activation of apoptosis processes via LC3B on MCF‐7 cells. In conclusion, we are thought that 5‐FUD‐Man has potential for use in breast cancer treatment.

## Author Contributions

**Ebru Şanci:** writing – original draft, methodology, formal analysis. **Azada Aliyeva:** methodology, formal analysis. **Buket Bakan:** methodology, formal analysis, data curation. **Mustafa Özkaraca:** methodology, formal analysis, data curation. **Erkan Halay:** methodology, formal analysis. **Kadir Ay:** methodology, formal analysis. **Tamer Karayildirim:** methodology, formal analysis, data curation. **N. Ulku Karabay Yavasoglu:** writing – reviewing draft, supervision, project administration, methodology, formal analysis, data curation. All authors read and approved the manuscript.

## Conflicts of Interest

The authors declare no conflicts of interest.

## Data Availability

The data that support the findings of this study are available from the corresponding author upon reasonable request.
